# Treatment of vitiligo with fire needle: A protocol for systematic review and meta analysis

**DOI:** 10.1097/MD.0000000000024106

**Published:** 2021-01-08

**Authors:** Liu Mingqiang, Qiu Guirong, Wu Yunbo, Lan Hongrong, Du Xiaoyan

**Affiliations:** a Jiangxi University of Traditional Chinese Medicine; b Affiliated Hospital of Jiangxi University of Traditional Chinese Medicine, Nanchang, Jiangxi, Peoples Republic of China.

**Keywords:** fire needle, meta analysis protocol, vitiligo

## Abstract

**Background:**

We aim to study the treatment of vitiligo with fire needle.

**Methods:**

We will search PubMed, Embase, the Cochrane Library, the China National Knowledge Infrastructure, Chinese Science and Technology Periodical Database, Wanfang Database, and Chinese Biomedical Literature Database of randomized controlled trials beginning from their inception to August 2020. The primary outcomes is that Complex area of white spot and Percentage of Clinical Effectiveness will be accepted as the primary outcomes. Additional outcome is the safety assessment will be considered a secondary outcome. Two independent authors will based on the Cochrane system evaluation manual 5.1.0 version of randomized controlled trial bias risk assessment tool to evaluate the risk of bias among the final included studies. And we will use the RevMan 5.3 software to analysis data.

**Conclusion:**

This study will provide evidence to judge whether fire needle is an effective therapy for vitiligo.

**Inplasy registration number:**

INPLASY2020120012.

## 1 Introduction

Vitiligo is a kind of depigmentation skin disease characterized by the appearance of clear opalescent plaques. The main etiology and pathogenesis is not clear, the main point of view is oxidative stress, melanocyte self destruction, autoimmune, genetic factors, and so on.^[1]^ Traditional Chinese medicine called the disease as baibifeng disease, the main etiology is exogenous wind evil, liver and kidney deficiency, emotional disharmony, and so on.^[2]^ However, there is no effective medicine to treat vitiligo, mainly through external treatment. TCM external treatment has certain advantages for the treatment of vitiligo. Studies have shown that vitiligo patients in the world have reached 1% of the total population, and the incidence rate in China is 0.553%. Therefore, we can conclude that vitiligo patients in China have nearly about 8,000,000.^[3,4]^ Now the master treatment of Western medicine includes external use of hormone ointment, calcineurin inhibitors, immunosuppressive agents, NB-UVB, 308 nm excimer light and other,^[5]^ but the treatment effect is not very reasonable. Yes. Vitiligo caused by skin pigmentation changes, not only affect the beauty of patients, but also seriously affect the psychological and quality of life of patients, but now the effect of treatment is not very ideal, increasing the medical burden of patients and weakening the confidence in treatment. At present, the fire needle which is widely used in the external treatment of traditional Chinese medicine has a certain effect on the treatment of vitiligo, but there is a lack of systematic analysis on the treatment of vitiligo with fire acupuncture. Therefore, the author makes a systematic analysis on the treatment of vitiligo from the perspective of efficacy and safety, hoping to provide basis for clinical treatment.

## 2 General information and methods

### 2.1 Types of study

All randomized controlled trials (RCTs) of fire needle therapy for vitiligo will be included without language restriction. Non-RCTs, observational studies, cross-over studies, uncontrolled trials, animal trials, and reviews will be excluded.

#### 2.1.1 Types of participants

Inclusion criteria for study populations will be all patients with vitiligo. No restrictions will be applied in terms of gender, age, race, condition duration, or intensity. The language is limited to Chinese and English.

#### 2.1.2 Types of interventions

##### 2.1.2.1 Experimental interventions

The treatment group will receive fire needle therapy and can be combined with external application of drugs and narrow-band violet External line, 308 excimer light, traditional Chinese medicine, and other methods, without any restrictions on needle material, shape, or treatment process.

##### 2.1.2.2 Control interventions

The control group will receive an internationally recognized therapy such as external application of drugs and narrow-band violet External line, 308 excimer light, Placebo, no treatment, but not use fire needle treatment;

#### 2.1.3 Types of outcome measures

##### 2.1.3.1 Primary outcomes

Complex area of white spot and Percentage of Clinical Effectiveness will be accepted as the primary outcomes.

##### 2.1.3.2 Additional outcomes

The safety assessment will be considered a secondary outcome.

### 2.2 Search methods for the identification of studies

#### 2.2.1 Electronics searches

The following electronic databases will be searched: PubMed, Embase, the Cochrane Library, the China National Knowledge Infrastructure, Chinese Science and Technology Periodical Database, Wanfang Database, and Chinese Biomedical Literature Database. We will search the databases from the beginning to AUGUST 2020. Search terms consist of disease (vitiligo, leucoderma, leukodermia) and intervention (fire needle, improved fire needle) and research types (randomized controlled trial, controlled clinical trial, random trials). The PubMed search strategy is shown in Table 1.

**Table 1:** Search strategy used in PubMed database.

Number	Searching item
1	vitiligo
2	leucoderma
3	leukodermia
4	1 or 2 or 3
5	Fire needle
6	Improved fire needle
7	5 or 6
8	randomized controlled trial
9	controlled clinical trial
10	random trials
11	8 or 9 or 10
12	4 and 7 and 11

#### 2.2.2 Search for other resources

We will also retrieve the relevant conference papers, and search for new trials related to fire needle of vitiligo on the World Health Organization International Clinical Trials Registration Platform and the Clinical Trials.gov.

### 2.3 Data collection and analysis

#### 2.3.1 Selection of studies

**Figure 1:**
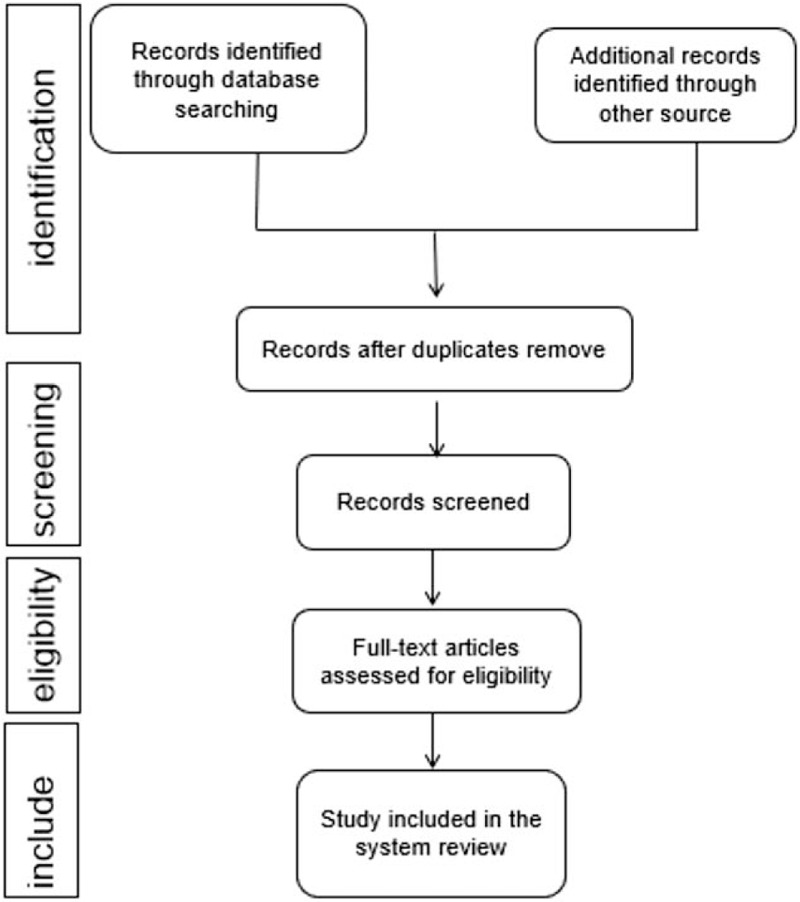
Flow diagram of study selection process.

We will import the retrieved literature into EndNote X9 software and delete the duplicate data. After that, 2 reviewers will independently scan the titles and abstracts. Unrelated literature will be deleted. If they cannot determine whether to include the study, they will obtain the full text of the article for judgment. Two reviewers will independently evaluate the eligibility of these articles based on inclusion and exclusion criteria. Any disagreements will be resolved through group discussions. The study selection procedure is shown in Figure 1.

#### 2.3.2 Data extraction and management

The data extraction for eligible studies will be completed independently by 2 authors, and any disagreement will be resolved through discussion with the third author. The extracted data will mainly include the first author, time of publication, patient characteristics, sample size, interventions, follow-up period, outcome measures, and adverse events. If necessary, we will try to contact the author for the details by email.

### 2.4 Risk of bias assessment

Two independent authors will based on the Cochrane system evaluation manual 5.1.0 version of RCT bias risk assessment tool^[6]^ to evaluate the risk of bias among the final included studies, the evaluation contents include:

(1) randomized controlled trials;
(2) whether allocation concealment is implemented;
(3) whether the experiment is blind;
(4) whether the evaluation of outcome indicators is blind;
(5) whether the outcome indicators are complete;
(6) whether selective reporting;
(7) whether there are other bias risks.

If there is ambiguity in the analysis, a third party will be added for negotiation.

### 2.5 Quantitative data synthesis and statistical methods

#### 2.5.1 Quantitative data synthesis

We will conduct statistical analysis through RevMan 5.3 software. For categorical data, we will calculate with the risk ratio and 95% confidence intervals. For continuous variables, mean difference will be included in the meta-analysis. If outcome variables are measured on different scales, results will be reported as standardized mean differences with 95% confidence interval.

#### 2.5.2 Assessment of heterogeneity

We will use *χ*^2^ test and *I*^*2*^ test to evaluate the statistical heterogeneity. When *P* > .10 and *I*^*2*^ ≤ 50%, the research results will not be considered heterogeneous; otherwise, it will be considered as heterogeneous.

#### 2.5.3 Assessment of reporting biases

When more than 10 studies are included, funnel plot will be generated to detect the reporting bias. In addition, we will use the Egger test to check the asymmetry of funnel plot.

#### 2.5.4 Subgroup analysis

If the included studies have significant heterogeneity, we will perform subgroup analysis based on different control groups.

#### 2.5.5 Sensitivity analysis

When sufficient studies are available, sensitivity analysis will be used to assess the robustness of the meta-analysis based on methodological quality, sample size, and missing data.

#### 2.5.6 Grading the quality of evidence

We will assess the quality of evidence by the Grading of Recommendations Assessment, Development and Evaluation and rate it into high, moderate, low, or very low 4 levels.^[7,8]^

## 3 Discussion

Vitiligo is a kind of Chinese medicine vitiligo, which is mainly caused by the disharmony of Qi and blood and blood stasis. The pathogenesis of Western medicine is mainly caused by the inhibition of tryptophan enzyme system and the decrease of melanocytes. Fire needle originated from the “burnt needle” and “Jue needle” in Huangdi Neijing, which laid the theoretical foundation of fire needle. After that, fire needle gradually developed and improved its indications and contraindications. It has developed to the application of today's fire needle. It integrates the functions of fire needle and filiform needle, and reduces the pain of patients.^[9]^ Traditional Chinese medicine believes that fire needle has the function of acupuncture and moxibustion. On the one hand, it can supplement the Yang Qi of the human body. By stimulating the meridians, regulating the function of viscera, and helping the healthy qi of the human body, it can promote the circulation of local Qi and blood, increase the local nutritional supply, thus stimulating the system of neuraminidase and increasing the activity of melanocytes This achieves the purpose of treatment.^[10]^ It is mentioned in Lingshu that “white is cold”. The cold nature is collected and induced, and the skin is blocked by depression, then Qi and blood cannot nourish the skin, and the skin will produce white spots. The fire needle takes its warm function to dispel pathogenic factors and promote the operation of Qi and blood.^[11]^ Modern medicine has found that the therapeutic effect of fire needle is equivalent to the effect of skin i-shallow II degree burn, which can increase the permeability of local capillaries, increase the permeability of blood vessels to tissues, and enhance the stress of the body. Therefore, the treatment of fire needle is inseparable from the immune function of skin.^[12]^ Lu Weiwei^[13]^ found that the mechanism of fire needle is the mechanism of damage and repair, especially after the second week of fire needle treatment, the repair function reaches the peak, enhances the activities of various enzymes, speeds up the metabolism of local tissues, can quickly absorb burned tissues, and promote the growth of healthy tissues. Some animal studies have found that the warm stimulation of fire needle and local tissue damage can increase the number of melanoblasts in hair follicles through the production of cytokines, which has reached the therapeutic effect.^[14]^ The warm effect of fire needle can stimulate the operation of local Qi and blood, activate the system of tryptophan enzyme, and increase the number of melanocytes; as well as damage and repair function. After the slight skin injury caused by fire needle, the skin regeneration mechanism can be used to accelerate tissue metabolism and promote the growth of new healthy tissue.

## Author contributions

**Conceptualization:** Liu Mingqiang, Qiu Guirong.

**Data curation:** Liu Mingqiang, Du Xiaoyan, Lan Hongrong.

**Formal analysis:** Liu Mingqiang, Wu Yunbo.

**Funding acquisition:** Liu Mingqiang, Wu Yunbo.

**Investigation:** Liu Mingqiang, Lan Hongrong.

**Methodology:** Liu Mingqiang, Wu Yunbo, Qiu Guirong.

**Project administration:** Liu Mingqiang.

**Resources:** Liu Mingqiang, Du Xiaoyan.

**Software:** Liu Mingqiang.

**Supervision:** Liu Mingqiang, Qiu Guirong.

**Validation:** Liu Mingqiang.

**Visualization:** Liu Mingqiang, Qiu Guirong.

**Writing – original draft:** Liu Mingqiang.

**Writing – review & editing:** Liu Mingqiang, Qiu Guirong.
